# Mechanisms Underlying the Cardioprotection of YangXinDingJi Capsule against Myocardial Ischemia in Rats

**DOI:** 10.1155/2020/8539148

**Published:** 2020-11-17

**Authors:** Miaomiao Liu, Yurun Xue, Yingran Liang, Yucong Xue, Xue Han, Ziliang Li, Li Chu

**Affiliations:** ^1^School of Pharmacy, Hebei University of Chinese Medicine, Shijiazhuang 050200, Hebei, China; ^2^Hebei Higher Education Institute Applied Technology Research Center on TCM Formula Preparation, Shijiazhuang 050091, China; ^3^School of Basic Medicine, Hebei University of Chinese Medicine, Shijiazhuang 050200, Hebei, China; ^4^Hebei Key Laboratory of Integrative Medicine on Liver-Kidney Patterns, Shijiazhuang 050200, Hebei, China

## Abstract

**Background:**

YangXinDingJi (YXDJ) capsule is one of traditional Chinese medicines (TCMs) derived from Zhigancao decoction, which is usually used for the treatment of cardiovascular disease in China. *Aim of the Study*. Cardiovascular events are one of the leading causes of death worldwide. Myocardial ischemia (MI) severely reduces myocyte longevity and function. The YangXinDingJi (YXDJ) capsule has been used in the treatment of clinical cardiac disease in China. Nevertheless, the underlying cellular mechanisms for the benefits to heart function resulting from the use of this capsule are still unclear. The aim of this study was to evaluate the protective effects of the YXDJ on isoprenaline-induced MI in rats and to clarify its underlying myocardial protective mechanisms based on L-type calcium channels and myocardial contractility.

**Materials and Methods:**

Rats were randomly divided into five groups with ten rats in each group: (1) control; (2) ISO-induced model; (3) high-dose YXDJ (2.8 g/kg/day intraperitoneally for five days), (4) low-dose YXDJ (1.4 g/kg/day for five days); and (5) verapamil (*n* = 10 in each group). Isoproterenol (ISO) was injected subcutaneously for two consecutive days to induce the rat model of MI. Heart and biochemical parameters were obtained. The patch-clamp technique was used to observe the regulatory effects of YXDJ on the L-type calcium current (I_Ca-L_) in isolated cardiomyocytes. An IonOptix MyoCam detection system was used to observe the contractility of YXDJ on isolated cardiomyocytes.

**Results:**

YXDJ caused a significant improvement in pathological heart morphology and alleviated oxidative stress and inflammatory responses. Exposure to YXDJ caused a decrease in blockade of I_Ca-L_ in a concentration-dependent manner.

**Conclusions:**

The results indicate that YXDJ significantly inhibited inflammatory cytokine expressions, oxidative stress, and L-type Ca^2+^ channels, and decreased contractility in isolated rat cardiomyocytes. These findings may be relevant to the cardioprotective efficacy of YXDJ.

## 1. Introduction

As a fatal disease, ischemic heart disease (IHD) has become one of the most serious health problems both in developing and developed countries [1, 2]. Prolonged ischemia can lead to reperfusion injury to cardiac myocytes, which may include varying degrees of cell death and myocardial edema [3]. Several factors are known to increase the risk for myocardial ischemia (MI); for example, beta-adrenergic stimulation is thought to exacerbate ongoing myocardial infarction [4]. Isoprenaline (ISO), a nonselective *β*-adrenoceptor agonist, is widely used to induce MI in experimental rat models of cardiac disease. In these models, activation of the adrenergic system causes severe stress in the myocardium by inducing an increase in the L-type Ca^2+^ channel (LTCC) activity [5].

In the occurrence and maintenance of MI, a number of mechanisms are involved that cause an imbalance of intracellular Ca^2+^. LTCCs are the main routes for calcium entry into cardiac myocytes, which is responsible for initiating contraction in the heart [6, 7]. LTCCs (also known as voltage-gated calcium channels, Cav_1.2_ channels, and dihydropyridine receptors) can respond to small changes in membrane potential [8]. This allows LTCC to create a “calcium window” that provides a small, sustained influx of Ca^2+^ into the cell that can activate the contractile mechanism [9]. Membrane depolarization via the action potential leads to the opening of L-type channels, which leads to heart contractions during systole [10]. Plasma membrane influx of Ca^2+^ by LTCCs leads to Ca^2+^-induced Ca^2+^ release, which in turn regulates cardiac contractility. Oxidative stress can cause some cellular defects such as decreasing of the sarcolemmal Ca^2+^ ATPase pump and Na^+^-K^+^ ATPase activities. These alterations lead to a decrease in the Ca^2+^ effluxes and an increase in the Ca^2+^ influxes, respectively [11].

Studies on the association of Ca^2+^ increase with an increase in oxidative stress have been contradictory, and the sequence of events remains controversial. The increase in cytosolic Ca^2+^ concentration is induced by reactive oxygen species (ROS) [12]. An increase in Ca^2+^ is a constant feature of pathological states associated with oxidative stress [13]. It has been found that myocardial ischemia is related to inflammatory response and oxidative stress [14, 15]. Oxidative stress is an imbalance between ROS and antioxidants and is actually the imbalance between the generations of ROS and body antioxidant defense systems that associated with many noncommunicable diseases, such as cancer, heart disease, and diabetes [16]. Excessive ROS causes oxidative damage to important cellular components such as DNA, protein, and lipid membranes. In MI, hypoxia and reoxygenation occur, which may induce the excessive production of ROS in cardiac tissues [17]. Free radicals are typically scavenged by antioxidant enzymes including superoxide dismutase (SOD) and malondialdehyde (MDA), and previous studies have suggested that the antioxidant activities of these enzymes may be reduced in patients following MI or in those with ischemic heart disease [18, 19]. After an MI, inflammatory response occurs, and macrophage infiltration gradually increases. In addition, resent research suggests that MI injury is a result of an increased expression of inflammatory cytokines [20]. Expression and release of inflammatory cytokines such as interleukin (IL)-6, tumor necrosis factor (TNF)-*α*, and several chemokines contribute to upregulation of cell-adhesion molecules, cardiac functional depression, and apoptosis [21].

Traditional Chinese medicine (TCM) has been widely used in clinical treatment of heart disease in China [22]. Zhigancao decoction is a TCM that originated from “*shang han lun*” written by Zhang Zhongjing in the Eastern Han Dynasty [23]. The therapeutic effects of Zhigancao decoction medicated serum are comparable to routinely used antiarrhythmic medicines. Long-term use in patients has also demonstrated good tolerance to the drug in patients [24]. YangXinDingJi capsule (YXDJ) evolved from the Zhigancao decoction, which is composed of *Radix ophiopogonis*, *Rehmannia*, licorice, ginger, red ginseng, jujube, donkey hide gelatin, black sesame, and cinnamon twig (see Table 1). This prescription was approved by the China Food and Drug Administration (license no. Z19991082) and is used in the treatment of palpitations caused by heart syndrome. According to previous research, the main constituents in YXDJ capsule, including liquiritin, glycyrrhizic acid, cinnamic acid, and cinnamic aldehyde, were analyzed by the HPLC analysis method using a Diamonsil C18 column and the results showed good linear relationships. The ranges of liquiritin, glycyrrhizic acid, cinnamic acid, and cinnamic aldehyde were 1.00–80.24 *μ*g/mL (*r* = 0.9990), 2.52–100.70 *μ*g/mL (*r* = 0.9997), 0.50–40.40 *μ*g/mL (*r* = 1.0000), and 0.66–52.96 *μ*g/mL (*r* = 1.0000), respectively [25]. A recent clinical trial confirmed its effectiveness for improving symptoms and reducing angina pectoris and complications along with few adverse effects [26]. However, the precise mechanism underlying the cellular Ca^2+^ homeostasis of YXDJ protects against cardiac remains poorly understood. We hypothesized that YXDJ plays a role in myocardial protection by regulating Ca^2+^ homeostasis, reducing oxidative stress, suppressing inflammatory cytokine expressions, and reducing cytoplasmic myocardial concentration.

In this paper, we evaluated the LTCC current (I_Ca-L_) changes with respect to relieving oxidative stress of YXDJ in cardiomyocytes. Further study on the cellular mechanisms of YXDJ will not only contribute to a better understanding of the efficacies in clinical treatments but also provide experimental evidence for rational applications.

## 2. Materials and Methods

### 2.1. Reagents

YangXinDingJi capsule (batch no. 06720021241, 0.5 g/capsule, approval number: Z19991082) was acquired from Yongfeng Pharmaceutical Co., Ltd. (Shijiazhuang, China). ISO was acquired from Amylet Scientific Inc. (Michigan, USA). Verapamil was acquired from Hefeng Pharmaceutical Co., Ltd. (Shanghai, China). Unless otherwise stated, other chemical reagents were obtained from Sigma-Aldrich (St. Louis, MO, USA). All solvents used were of analytical grade.

### 2.2. Animals

A total of 100 adult male Sprague-Dawley rats weighing 200 ± 20 g are used in this study. The rats were provided by the Experimental Animal Center of Hebei Province. Thirty-eight rats were used for patch clamp to record Ca^2+^ currents, twelve rats were used to detect cell shortening, and fifty rats were used for experiment in vivo. Male adult SD rats (200 ± 20 g) were exposed to an environment of 22 ± 2°C, and humidity was 55 ± 5% with a 12 h light-12 h dark cycle and adequate food and water supplies. All studies were performed in conformity with the guidelines for care and standard experimental animals' study ethical protocols and approved by the Ethics Committee for Animal Experiments of Hebei University of Chinese Medicine.

### 2.3. Induction of Myocardial Ischemia Injury

After acclimatization to laboratory conditions, fifty male SD rats were randomly assigned into five experimental groups (*n* = 10 per group): (1) control group (CON); (2) ISO-induction group (ISO); (3) verapamil treatment group (VER); (4) high-dose YXDJ (YXDJ_H_); and (5) low-dose YXDJ (YXDJ_L_). The CON and ISO groups were gavaged with distilled water. YXDJ_H_ and YXDJ_L_ groups were gavaged with YXDJ (2.8 and 1.4 g/kg/day, respectively). The VER group was intraperitoneally injected with verapamil (2 mg/kg/day). After seven days of continuous administration, rats were subcutaneously injected with ISO for two consecutive days (85 mg/kg/day, except in the CON group). At the end of the experiment, the rats were anesthetized with sodium pentobarbital (40 mg/kg) and hearts were removed and examined as described below.

### 2.4. Electrocardiogram

At the end of the experimental period, the anesthetized rats were examined by electrocardiogram (ECG). The needle electrodes were linked to the rat's right arm, front arms, and left leg. ECGs were recorded with needle electrodes and a BL-420S Biological Data Acquisition & Analysis System (Chengdu TEM Technology Co., Ltd., China).

### 2.5. Myocardium Histopathology

The animal hearts were dissected rapidly and fixed in 4% paraformaldehyde. The heart tissues obtained from rats were embedded in paraffin and sectioned into at 4-*μ*m slices. The heart tissues were processed for sectioning and staining by standard histological methods. The results were observed under a microscope (Leica DM4000B, Solms, Germany).

### 2.6. Ultrastructural Examination by Transmission Electron Microscopy

Rats were anesthetized, and the hearts were quickly removed. Heart tissues were cut into small pieces (approximately, 1 mm^3^). These pieces were then rapidly fixed with 2.5% glutaraldehyde in 0.1 mol/L sodium phosphate buffer (pH 7.4) at 4°C for 3 h and then fixed again with 1% osmium tetroxide at 4°C for 1 h. After dehydration with a graded ethanol series, the thin sections were cut with a Leica EM UC6 (Leica Co, Austria) ultramicrotome. A sample was embedded in epon812, and the section was viewed and photographed on a Hitachi 7650 transmission electron microscope (TEM) from (Hitachi, Japan) at 80 kV.

### 2.7. Serum Preparation for Detection of CK, LDH, SOD, and MDA

Blood was obtained from rats that had been anesthetized at the end of 7 days of treatment. Serum was pipetted and saved at −20°C following centrifugation at 3000 rpm at 4°C for 10–15 min. CK and LDH, SOD activity, and MDA levels were determined by standard commercial kits (Jiancheng, Nanjing, China) according to the manufacturer's instructions.

### 2.8. Detection of Intracellular ROS

Dihydroethidium (DHE) oxidation is commonly used as a method for monitoring cellular production of ROS. The fluorescence of DHE probe was used to evaluate ROS production in heart tissues. We studied quantitative changes in DHE oxidation products. ROS generation was labeled with the red fluorescence, and visualized and analyzed using a high-content screening system. All experiments were repeated at least three times.

### 2.9. Inflammation Assay

After centrifugation, serum was collected. IL-6 and TNF-*α* levels in serum were determined to examine the relationship between the cardioprotective effects of YXDJ and inflammatory cytokine levels. Serum level of IL-6 was calculated from the kit standards and expressed as pg/mL, and TNF-*α* was expressed as pg/mL.

### 2.10. Detection of Calcium Concentration in Myocardial Tissues

The Ca^2+^ concentration was determined in heart tissue. Myocardial tissues were placed in a buffered solution (m/V = 1 : 9). The homogenate was centrifuged at 1000 rpm at 4°C for 10 min to remove cell debris, and the supernatant was saved for all downstream biochemical analyses. Ca^2+^ concentrations were evaluated by Coomassie Brilliant Blue staining with a commercial kit (Jiancheng, Nanjing, China).

### 2.11. Isolation of Cardiomyocytes

Single myocardial cells were obtained via enzymolysis. Rats were intraperitoneally injected with 500 IU/kg heparin and anesthetized with 40 mg/kg sodium pentobarbital. The heart was quickly removed from the rat, placed on a Langendorff instrument for perfusion with an oxygenated free calcium Tyrode's solution, which contained (mM) NaH_2_PO_4_ 0.33, NaCl 135, KCl 5.4, glucose 10, MgCl_2_ 1.0, and HEPES 10 (pH adjusted to 7.4 with 3 M NaOH). After clearance of the blood, the enzyme solution containing 4 g/L taurine, 10 g/L bovine serum albumin (Roche, Basel, Switzerland), and 4 g/L collagenase type II (GIBCO, Invitrogen, Carlsbad, CA, USA) was perfused for 15 to 25 min at 37°C until the heart was soft. After perfusion, the heart was placed into Krebs buffer (KB) and the heart tissue was dissected into small pieces. The cardiomyocytes were preserved in KB solution (bubbled with O_2_) at a temperature of 23–25°C for up to 1 to 2 h before the experiment.

### 2.12. Electrophysiological Techniques

Inward Ca^2+^ currents were recorded from rat myocytes. Whole-cell L-type Ca^2+^ currents were recorded using the conventional patch-clamp technique, as previously described [27]. Pipettes of 4 to 6 MΩ resistance were pulled from borosilicate glass (Kimax 51, KIMBLE Glass Inc.) Microelectrodes had resistances of 4 to 6 MΩ and were lightly fire polished and filled with an intracellular solution containing MgCl_2_ 2.0 mM, glucose 10 mM, CaCl_2_ 1.8 mM, TEA-Cl 140 mM, HEPES 10 mM, and TTX 10 *μ*M (pH 7.4). Seventy to ninety percent of the series resistance was compensated. Cells were bathed in a potassium-free extracellular solution that contained (in mM) 140 NaCl, 5.4 CsCl, 2.5 CaCl_2_, 0.5 MgCl_2_, 11 glucose, and 5.5 HEPES (pH 7.4). YXDJ capsule was dissolved in distilled water before, and then dissolved in extracellular solution when patch clamp is used to recorded Ca^2+^ currents at the following concentration of 100–500 *μ*g/mL. Whole-cell patch-clamp experiments were carried out at room temperature (20–25°C). For all experiments, the holding potential was −80 mV and currents were elicited by a 200-msec depolarizing pulse. Data were analyzed using the pCLAMP (Axon Instruments, Inc.).

### 2.13. Measurements of Cell Shortening

Ventricular myocytes were placed at the bottom of the glass chamber, and the normal Tyrode's solution flowed at a speed of 1 mL/min. Cell activity could be observed with an inverted microscope and a detection system placed on the glass chamber. Cell shortening at 500 *μ*g/mL of YXDJ was measured to determine the effects of YXDJ on myocardial contractility. Myocytes with clear edges were selected to measure cell shortening before and after MI. The contractions of ventricular myocytes were assessed with an IonOptix MyoCam detection system (IonOptix, Milton, MA, USA).

### 2.14. Statistical Analysis

The steady-state activation and inactivation curves of I_Ca-L_ were fitted by Boltzmann functions. These conductances were normalized to their individual maximal conductance. All the values are represented as mean ± standard error of the mean (SEM) and were analyzed by one-way analysis of variance (ANOVA). The statistical differences among different groups were analyzed by Student's *t*-test. *P* values of <0.05 were considered significant.

## 3. Result

### 3.1. Effects of YXDJ on Electrocardiography

**Figure 1:**
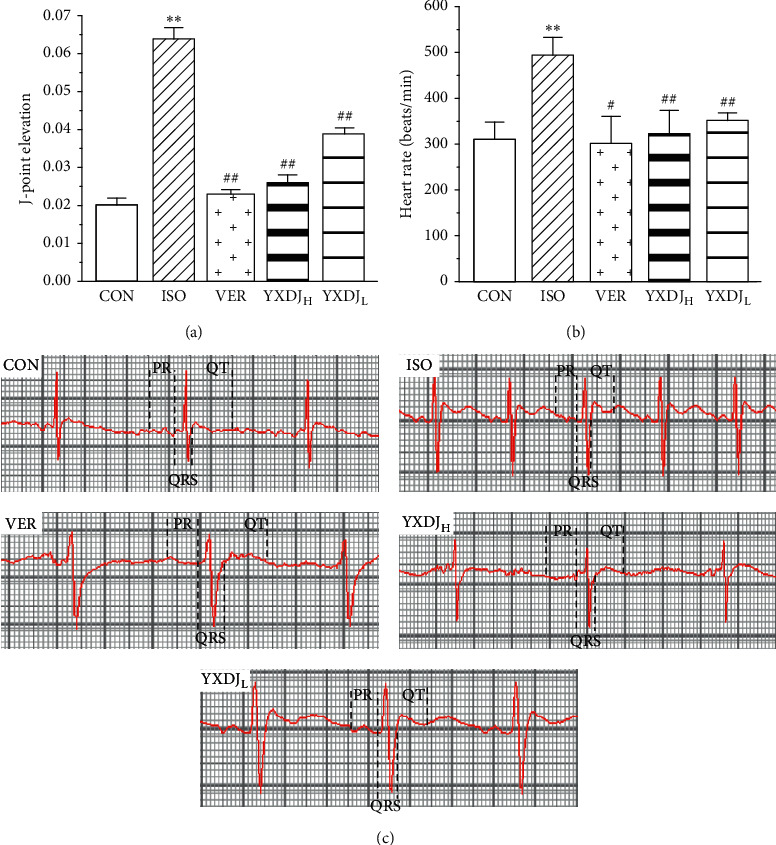
Actions of YangXinDingJi (YXDJ) on the electrocardiogram (ECG): (a) statistical analysis of J-point elevation, (b) heart rate, and (c) representative ECG tracings. Values represent the mean ± SE, for 10 rats in each group. ^*∗∗*^*P* < 0.05 versus control (CON); ^#^*P* < 0.05, ^##^*P* < 0.01 versus isoproterenol (ISO).

Analysis of electrocardiography patterns was done to determine the actions of YXDJ on ISO-induced MI animals. As shown in Figures 1(a) and 1(b), the obvious reduction in the J-point elevation and heart rate was achieved in the YXDJ groups at both the high and low doses compared to ISO (*P* < 0.05; *P* < 0.01). Compared with CON, the heart rate and J-point of ISO-induced rats were evidently elevated.

Figure 1(c) shows sample ECG tracings from the experimental rats. The ECG of the CON group was normal. In the ISO group, the amplitude of the R wave decreased and the ST interval increased, suggesting that ISO induced a reduction in the amplitude of ECG waves and an increase in frequency. The YXDJ group and VER group showed a recovered R wave amplitude and ST interval.

### 3.2. Effects of YXDJ on the Pathological Changes of Rat Hearts

**Figure 2:**
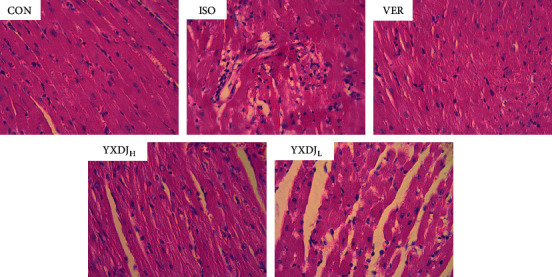
Effects of YXDJ on hematoxylin and eosin (H&E) staining. Representative microscopic photographs of hearts stained with H&E (magnification: 400x). The myocardial tissues obtained from the CON, ISO, verapamil (VER), and high- and low-dose YXDJ groups. Scale bar: 50 *μ*m.

The biochemical alterations mentioned above could be correlated with the histological changes in the heart, as shown in Figure 2. According to the hematoxylin and eosin (H&E) staining of the heart tissue, the heart tissue showed the normal histology of the CON group, which was composed of a natural muscle fibril structure. However, H&E staining showed that ISO-induced structural alterations in the myocardium in which cardiomyocytes appeared smaller, cardiac muscle fiber was disorganized, and vestigial infiltrating inflammatory cells were present. The damage in the YXDJ and VER administration groups was lower than that in the ISO group.

### 3.3. Effects of YXDJ on the Myocardial Mitochondrion Ultrastructure

**Figure 3:**
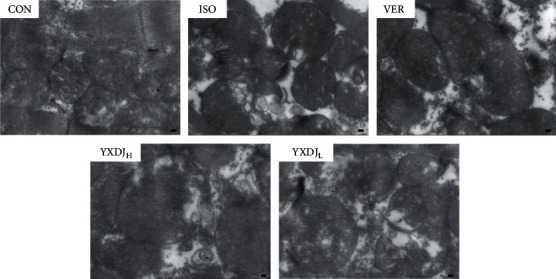
Actions of YXDJ on ultrastructural changes. The ultrastructure of cardiac tissues was detected by TEM from the ventricle of the CON, ISO, VER, and high- and low-dose YXDJ groups (magnification: 15000x). Scale bar: 1.0 *μ*m.

Mitochondrial ultrastructure was observed by TEM. As shown in Figure 3, the ultrastructure of the cardiac muscle from rats of the CON and VER groups was identical. However, myocardial mitochondria from the ISO group suffered substantial structural damage, including disordered mitochondrial distribution with disarranged and obscure crista and vacuoles within the matrix accompanying by disrupted sarcomere and myofilament. Noticeably, YXDJ pretreated alleviated these deleterious effects of mitochondrial ultrastructure induced by MI injury.

### 3.4. Effects of YXDJ on Cardiac Marker Enzymes

**Figure 4:**
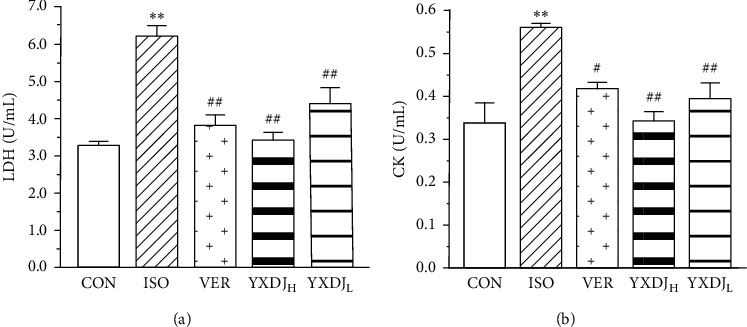
Actions of YXDJ on cardiac marker enzymes. Sera were analyzed for (a) lactate dehydrogenase (LDH) and (b) creatine kinase (CK). Values represent the mean ± SE, for 10 rats in each group. ^*∗∗*^*P* < 0.05 versus CON; ^#^*P* < 0.05, ^##^*P* < 0.01 versus ISO.

Cardiac markers in blood are the mainstay for cardiac damage diagnosis. As shown in Figure 4, a significant increase in cardiac markers was noted after ISO induction (*P* < 0.05) compared with the CON group. The serum CK and LDH activities of rats in the VER- and YXDJ-treated groups were significantly lower than those in the ISO group (*P* < 0.05, *P* < 0.01).

### 3.5. Effects of YXDJ on SOD and MDA in Serum

**Figure 5:**
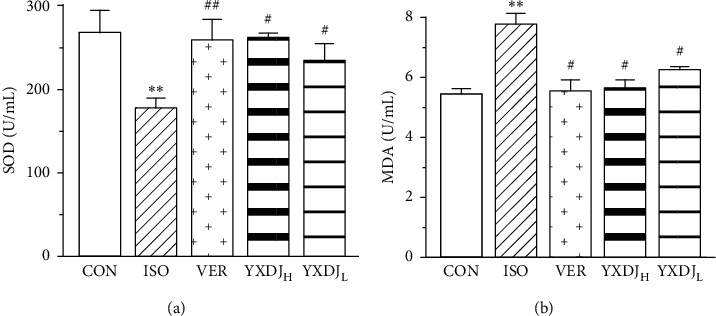
Actions of YXDJ on the superoxide dismutase (SOD) activity (a) and malondialdehyde (MDA) levels (b). Values represent the mean ± SE, for 10 rats in each group. ^*∗∗*^*P* < 0.05 versus CON; ^#^*P* < 0.05, ^##^*P* < 0.01 versus ISO.

To investigate the antioxidative effects of YXDJ, the action of YXDJ on ISO-induced SOD activity and MDA levels were detected. The activity of SOD in serum, as shown in Figure 5, ISO group rats was markedly lower than that of the CON rats (*P* < 0.05). However, after treatment with YXDJ (2.8 and 1.4 g/kg/day) and VER, the activity of SOD evidently increased (*P* < 0.05; *P* < 0.01). MDA content was significantly higher in the ISO group than in the CON group (*P* < 0.05). Meanwhile, compared with the ISO-induced rats, the MDA levels significantly reduced after treatment with YXDJ (2.8 and 1.4 g/kg/day) and VER (*P* < 0.05).

### 3.6. Effects of YXDJ on ROS Release

**Figure 6:**
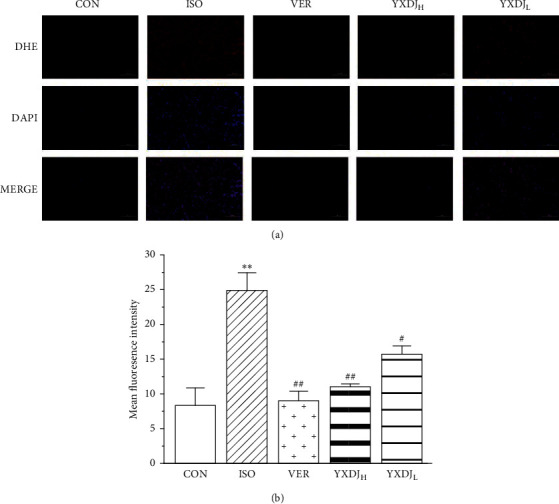
Actions of YXDJ on production of reactive oxygen species (ROS). Representative sections from the cardiac of the CON, ISO, VER, and high- and low-dose YXDJ groups. ROS generation was monitored by dihydroethidium staining. Values represent the mean ± SE. ^*∗∗*^*P* < 0.05 versus CON; ^#^*P* < 0.05, ^##^*P* < 0.01 versus ISO. Scale bar: 100 *μ*m.

The effect of YXDJ on the ISO-induced MI was evaluated with fluorescence of a dihydroethidium probe, specifically for assessing myocardial tissue ROS. As shown in Figure 6, VER obviously caused a reduction in the production of ROS. ROS evaluation revealed similar results in animals treated with YXDJ at both high and low does (*P* < 0.05; *P* < 0.01). The results implied that YXDJ could cause a reduction in ISO-induced oxidative stress.

### 3.7. Effects of YXDJ on IL-6 and TNF-*α*

**Figure 7:**
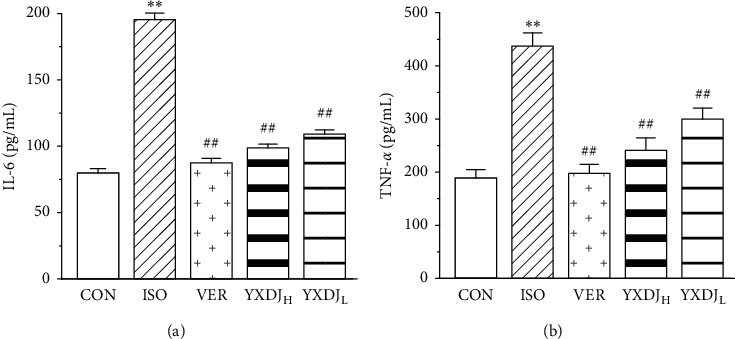
Effects of YXDJ on the expression of interleukin (IL)-6 and tumor necrosis factor (TNF)-*α* in ISO-induced myocardial infarction (MI): (a) the expression of IL-6 in serum; (b) the expression of TNF-*α* in serum. Values represent the mean ± SE, for 10 rats in each group. ^*∗∗*^*P* < 0.05 versus CON; ^#^*P* < 0.05, ^##^*P* < 0.01 versus ISO.

To further evaluate and validate the protective function of YXDJ during MI injury, we measured the levels of IL-6 and TNF-*α* in serum. The levels of TNF-*α* and IL-6 were significantly increased after ISO induction. Preprocessing by YXDJ caused a decrease in the expressions of IL-6 and TNF-*α* as shown in Figures 7(a) and 7(b) (*P* < 0.01). However, serum IL-6 and TNF-*α* concentrations in VER and high-dose YXDJ groups remained higher than those in the low-dose YXDJL group.

### 3.8. Effects of YXDJ on Calcium Concentration

**Figure 8:**
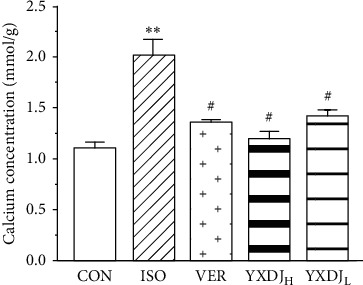
Actions of YXDJ on the Ca^2+^ concentrations. Values represent the mean ± SE, for 10 rats in each group. ^*∗∗*^*P* < 0.05 versus CON; ^#^*P* < 0.05, ^##^*P* < 0.01 versus ISO.

As shown in Figure 8, ISO induction of MI resulted in a significant increase in the Ca^2+^ concentration of the heart tissue relative to the CON. However, compared with the ISO group, the YXDJ group and VER group were significantly decreased (*P* < 0.05).

### 3.9. Reduction of I_Ca-L_ and Cell Shortening by YXDJ

#### 3.9.1. Confirmation of I_Ca-L_

**Figure 9:**
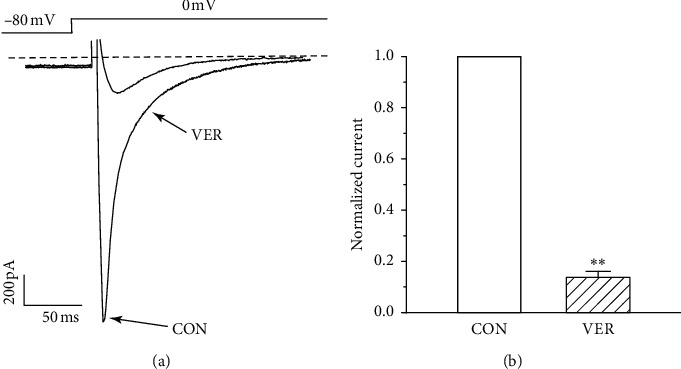
Confirmation of I_Ca-L_ in myocardial cells: (a) representative trace of after treatment by VER (0.1 mM); (b) summary data of (a). Values represent the mean ± SE, for 7 cells in each group. ^*∗∗*^*P* < 0.05 versus CON.

As shown in Figure 9, VER (0.1 mM), as a specific I_Ca-L_ blocker, caused an almost complete abolishment of the currents, indicating that these currents were Ca^2+^ currents (*P* < 0.01).

#### 3.9.2. Effects of YXDJ on I_Ca-L_ of Normal and Ischemic Myocyte Cell

**Figure 10:**
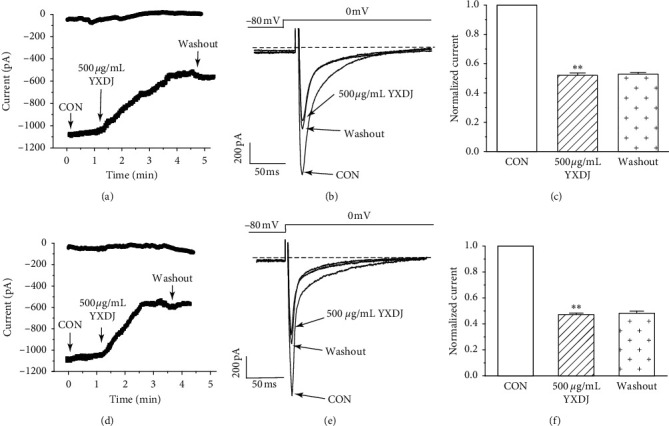
Actions of YXDJ capsule on I_Ca-L_ of healthy and ischemic cardiomyocytes. Actions of YXDJ capsule on I_Ca-L_ of healthy myocardial cells (a–c) and ischemic myocardial cells (d–f). YXDJ capsule I_Ca-L_ under control conditions, 500 *μ*g/mL YXDJ, and washout. Values represent the mean ± SE for 7 cells in each group. ^*∗∗*^*P* < 0.05 versus CON.

As Figures 10(a)–10(c) show, I_Ca-L_ decreased after treatment with 500 *μ*g/mL YXDJ with an inhibition rate of 52.0% in normal ventricular myocytes (*P* < 0.05). However, the I_Ca-L_ was almost impossible to recover after washing out with the external solution. As shown in Figures 10(d)–10(f), after the 500 *μ*g/mL YXDJ treatment, I_Ca-L_ was significantly reduced, and the inhibition rate was 47.21% in ischemic ventricular myocytes (*P* < 0.05); however, the I_Ca-L_ was almost impossible to recover, after washing out with the external solution.

#### 3.9.3. Dose Dependence of YXDJ on I_Ca-L_

**Figure 11:**
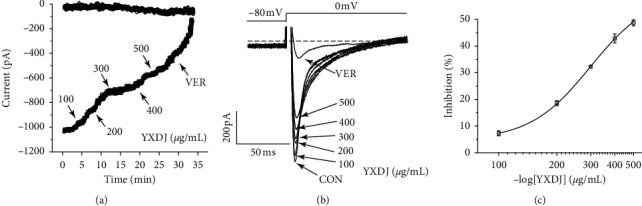
Actions of YXDJ capsule at different concentrations on I_Ca-L_: (a) time course of exposure to 100–500 *μ*g/mL (in 100 *μ*g/mL increments) and 0.1 mM VER; (b) example of current traces of I_Ca-L_ recorded during exposure to 100–500 *μ*g/mL and 0.1 mM VER; (c) concentration-response curve representing the percent inhibitory of YXDJ. Values represent the mean ± SE, for 7 cells in each group. ^*∗∗*^*P* < 0.05 versus CON.

The time course of the peak ICa-L was progressively decreased by increasing doses of YXDJ (100, 200, 300, 400, and 500 *μ*g/mL) or VER. As shown in Figure 11, different concentrations of YXDJ were used in the current traces induced by the test potential from −80 to 0 mV. The inhibition rates of YXDJ at 100–500 *μ*g/mL (in 100 *μ*g/mL increments) were 7.25% ± 0.94%, 18.57% ± 0.89%, 32.25% ± 0.47%, 44.63% ± 1.69%, and 48.75% ± 1.11%, respectively.

#### 3.9.4. Effects of YXDJ on I-V Relationship

**Figure 12:**
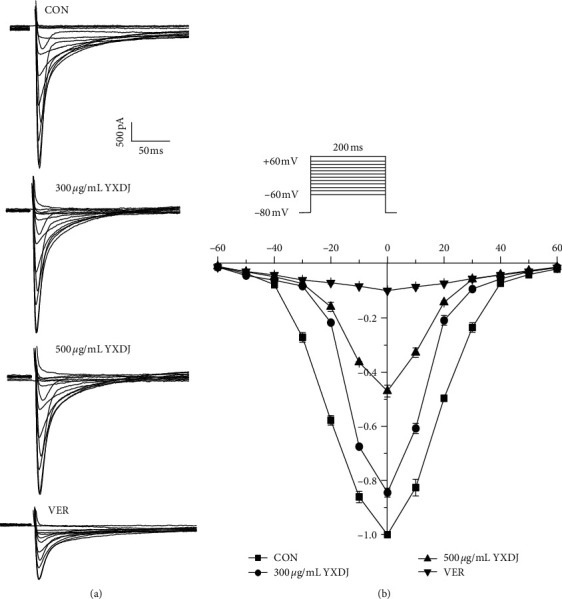
Actions of YXDJ capsule on the I–V relationship: (a) representative I_Ca-L_ current recordings and (b) the I–V relationship for I_Ca-L_ under the treatment of Con (□), YXDJ at 300 *μ*g/mL (○), YXDJ at 500 *μ*g/mL (△), and VER at 0.1 mM (▽). Values represent the mean ± SE for 7 cells in each group.

As shown in Figure 12, the effects of YXDJ (300 and 500 *μ*g/mL) and 0.1 mM VER on the I–V relationship of the I_Ca-L_ are depicted. The current was generated from −60 to 60 mV. After the application of YXDJ, I–V curves shifted upward, indicating that YXDJ has a concentration-/time-dependent effect on inhibition of I_Ca-L_. However, the peak potential of I_Ca-L_ and activated potential were markedly unchanged over the measured time period.

#### 3.9.5. Effects of YXDJ on Steady-State Activation and Inactivation of I_Ca-L_

**Figure 13:**
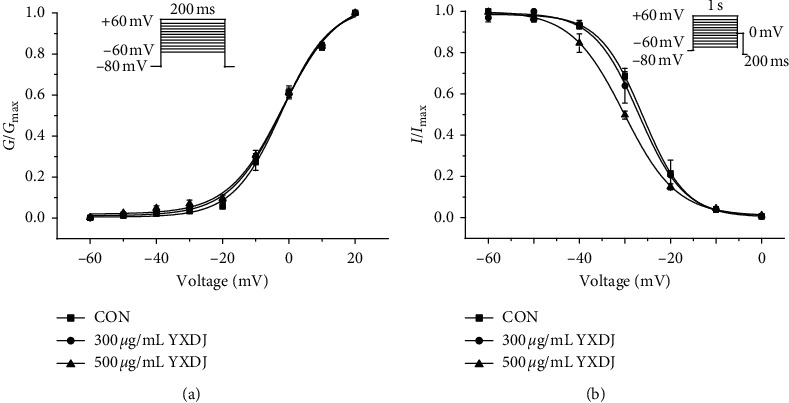
Actions of YXDJ capsule on steady-state activation and inactivation of I_Ca-L_: (a) steady-state activation curves and (b) steady-state inactivation of I_Ca-L_ are shown under the treatment of CON (□), YXDJ at 300 *μ*g/mL (○), and YXDJ at 500 *μ*g/mL (△). Values represent the mean ± SE for 7 cells in each group.

As shown in Figure 13, different concentrations of YXDJ (300 and 500 *μ*g/mL) affected steady-state activation and inactivation of I_Ca-L_. YXDJ at 300 and 500 *μ*g/mL did not change the inactivation and activation of I_Ca-L_. The *V*_1/2_ value for activation of 0, 300, and 500 *μ*g/mL YXDJ was −2.24 ± 0.87 mV/7.30 ± 0.73, −1.95 ± 0.77 mV/8.01 ± 0.63, and −1.82 ± 0.98 mV/8.25 ± 0.79, respectively. The *V*_1/2_ value for inactivation caused by 0, 300, and 500 *μ*g/mL YXDJ was −26.10506 ± 0.28256 mV/4.76285 ± 0.23397, −26.94475 ± 0.41879 mV/4.96567 ± 0.36614, and −30.21752 ± 0.12523 mV/5.77407 ± 0.1198, respectively. These data suggest that YXDJ did not alter the activation and inactivation of cardiac Ca^2+^ gating properties (*P* > 0.05). There were no significant differences between values of *V*_1/2_ in the presence and absence of YXDJ for the normalized inactivation and activation.

#### 3.9.6. Effects of YXDJ on Myocyte Shortening

**Figure 14:**
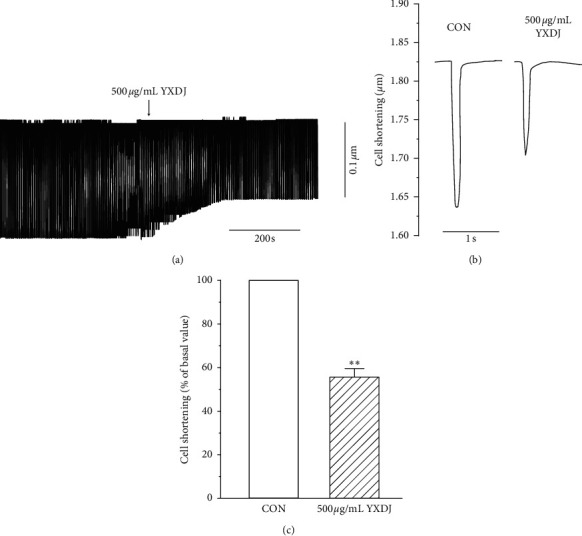
Actions of YXDJ capsule on cell shortening: (a) time parameters of cell shortening recordings; (b) exemplary traces of cell shortening under the CON and with 500 *μ*g/mL YXDJ; (c) summary results of (b). Values represent the mean ± SE for 7 cells in each group. ^*∗∗*^*P* < 0.05 versus CON.

The representative cell shortening recordings before and after administration of YXDJ are shown in Figure 14. The results indicate that 500 *μ*g/mL YXDJ caused a significant inhibition of cell shortening by 44.4% ± 3.89%.

## 4. Discussion

The goal for this study was to determine whether the YangXinDingJi capsule (YXDJ) was efficacious in protecting against MI by regulating calcium homeostasis. MI causes cardiac tissue damage via reduced levels of nutrients and oxygen due to a temporary decrease or shortage of blood supply. The cardiac muscle disease has partially been reported, and some authors proved an alteration of calcium homeostasis, especially in L-type voltage-gated calcium channel (VGCC) expression and regulation. TCM as therapy for MI has better prospects because of its components and targets [28].

In China, TCM has been considered an alternative and adjuvant approach for preventing cardiovascular disease (CVD) and ischemia injury [29, 30]. While, Zhigancao decoction is a Chinese herbal prescription generally used to treat arrhythmia and palpitation in clinics. YXDJ is a Chinese patent medicine, which was derived from Zhigancao decoction. It is used to treat heart disease in clinics. Although the capsule has been shown to be clinically effective, its protective mechanism on the heart is still unclear. In this paper, we investigated the regulation effect of calcium homeostasis and possible mechanism of YXDJ on ISO-induced MI. However, clinical studies are limited because of the difficulty in obtaining human myocardial samples from myocardial ischemia consumers. In experimental studies, myocardial ischemia models were replicated in animals comparable to what occurs in the human model.

Measurement of cardiac markers in blood is the mainstay for diagnosis of acute myocardial infarction [31]. Therefore, CK and LDH were used as biochemical indicators for detecting changes in ISO-induced MI. As shown in Figure 4, CK and LDH activities decreased after treatment with YXDJ. Meanwhile, as Figure 2 shows, ISO-induced histopathological changes in animal hearts showed that cardiomyocytes appeared smaller, cardiac muscle fibers were disorganized, and vestigial infiltrating inflammatory cells were present. After treatment with YXDJ and VER, histopathological sections show similarly normal structures, slight edema, a small number of inflammatory cardiomyocytes, and clear transverse fringes. The ultrastructure of the myocardium was also changed as shown in Figure 3. Myocardial mitochondria from the ISO group suffered substantial structural damage, and some mitochondria were fused, swollen, or cristae. However, the morphological changes in the YXDJ and VER groups showed significant improvements. In this study, the results of histopathology and cardiac marker enzymes showed that YXDJ caused a significant decreased in ISO-induced myocardial ischemic injury.

Isoprenaline (ISO) is a nonselective *β*-adrenoceptor agonist, and it is widely accepted that ISO injection can readily induce MI in animals [30, 32]. Stimulated *β*-adrenergic receptors may induce intracellular Ca^2+^ overload, inflammation, and accumulation of ROS leading to cardiac problems. In addition, the overloading of Ca^2+^ can induce arrhythmias and myocardial ischemia [33, 34]. Thus, blocking the Ca^2+^ channels and reducing the Ca^2+^ overload will benefit the treatment of arrhythmias and myocardial ischemic. Our study demonstrates that ISO induction caused MI and arrhythmias, which causes changes in ECGs, such as tachycardia and J-point elevation, while treatment with YXDJ could alleviate tachycardia and reduce J-point elevation. Therefore, YXDJ treatment can reduce the incidence of cardiac arrhythmias and MI in rats.

MI induced by ISO is mediated by vasospasticity, combined with increased oxygen demand due to its positive inotropic effect, and is related to the oxidative stress. Antioxidant activity is one of the key mechanisms of anti-MI efficacy. There are many kinds of free radicals, and ROS is closely associated with oxidative stress [35]. At the same time, some data have shown that Ca^2+^ activates the ROS-scavenging enzymes, such as SOD and MDA [36]. As shown in Figures 5 and 6, SOD activities, MDA concentrations, and intracellular ROS were detected. The oxidative stress injury in the ISO-induced group was more serious than other groups. After YXDJ treatment, the production of ROS in cardiomyocytes and MDA concentration were obviously reduced, and the activity of SOD was obviously increased. These results indicate that YXDJ can alleviate the oxidative stress response by causing improvements in SOD activity and MDA levels. Therefore, the mechanism of YXDJ in alleviating myocardial ischemic might be related to inhibition of oxidative stress-related reactions. The anti-MI properties of YXDJ were then assessed by studying different inflammatory markers. As shown in Figure 7, evaluation of inflammatory markers showed that ISO induced an obvious increase in TNF-*α* and IL-6 levels. Pretreatment with YXDJ and VER led to a decreased in the TNF-*α* and IL-6 levels, suggesting that the anti-inflammatory properties were associated with its cardioprotective effects of YXDJ.

Ca^2+^ is thought to play a significant role in controlling the prooxidant-antioxidant balance. An increase of cytosolic Ca^2+^ concentration is due to ROS, and Ca^2+^ increase is a constant feature of pathological states associated with oxidative stress [37]. We tested the effects of YXDJ on heart tissues and found that it caused a reduction in the calcium concentration. Therefore, the effects of YXDJ on Ca^2+^ channels and intracellular conditions were investigated using the whole-cell patch-clamp technique.

The results show that YXDJ led to a reduction in the I_Ca-L_. As shown in Figure 11, YXDJ caused a reduction in I_Ca-L_ in a concentration-dependent manner with an IC_50_ of 299.26 *μ*g/mL. Furthermore, I_Ca-L_ was also reduced by YXDJ at 500 *μ*g/mL in both ischemic ventricular and healthy ventricular myocytes. However, the I_Ca-L_ reverse potential and I–V relationship remained unchanged. The heart is an excitable organ, driven by excitation-contraction (EC) coupling that undergoes spontaneous force generation and relaxation cycles. A unique feature of EC coupling is the timely circulation of cytoplasmic calcium under control of a large group of proteins, including Na^+^/Ca^2+^ exchanger (NCX), phospholamban-regulated Ca^2+^-ATPase (SERCA), and ryanodine receptor (RyR) [38]. The process of Ca^2+^ induced Ca^2+^ release (CICR) is caused by Ca^2+^ inflow that triggers Ca^2+^ release from the sarcoplasmic reticulum (SR). However, CICR provides the necessary signal to initiate mechanical shortening of the cell contraction mechanism and can lead to several-fold increase in cytosolic Ca^2+^ concentration [39]. Nevertheless, the mechanisms for altering calcium homeostasis have still not been clearly established. The function of calcium channel antagonists is to reduce the entering rate of Ca^2+^ and turn off the calcium channel. Calcium antagonists are introduced into the therapeutic armory because of their specific effects on oxygen consumption, myocardial contractility and myocardial blood supply. In this present study, patch-clamp methods were used to examine excitation-contraction (EC) coupling, and we found that YXDJ had a significant inhibitory effect on I_Ca-L_. It seems that YXDJ could be used as a cardioprotective agent to inhibit I_Ca-L_ and reduce myocardial contractility. These results suggest that YXDJ caused the I_Ca-L_ inhibition primarily by reducing the Ca^2+^ current amplitude.

In addition, contractility was inhibited by YXDJ. This result can be explained by the process of myocardial contraction. Contraction is related not only to intracellular Ca^2+^ concentration but also to intracellular proteins that are involved in contraction. We utilized an IonOptix MyoCam detection system to examine the impact of YXDJ on rat ventricular myocytes. In this study, YXDJ significantly reduced the Ca^2+^ contractility, which may be related to the inhibition of I_Ca-L_. Furthermore, the present study focused on the effect of YXDJ in the of MI rats. To investigate the mechanism through which YXDJ exerts antiarrhythmic effect, it is necessary to study the related ion channels. However, the L-type Ca^2+^ channel is involved in the predominant mechanism responsible for the influx of Ca^2+^ in cardiac cells. We found that YXDJ can inhibit LTCC reducing calcium ions released, thereby reducing free intracellular Ca^2+^ and inhibiting cell shortening. This series of studies reveal that YXDJ can effectively reduce calcium overload and effectively relieve arrhythmias and myocardial ischemia.

Our data suggest that YXDJ could inhibit the increase in Ca^2+^ concentration via a decrease in the extracellular Ca^2+^ influx. Sufficient evidence links YXDJ to cardioprotection via inhibition of I_Ca-L_ and myocardial contractility.

## 5. Conclusion

**Figure 15:**
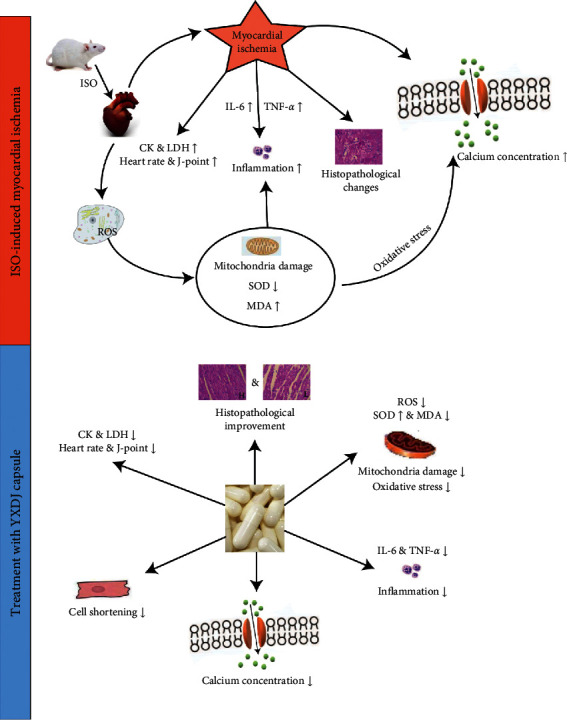
Cardioprotective effects of YXDJ capsule on ISO-induced MI.

The results demonstrated the cardioprotective effects of YXDJ in ISO-induced MI (see Figure 15). The mechanism may be related to regulating oxidative stress, anti-inflammatory effects, and reduction of calcium inflow via LTCC inhibition. These results provide the basis for further understanding the molecular mechanism of the beneficial effects of YXDJ on IHD.

## Figures and Tables

**Table 1:** Description of YangXinDingJi capsule.

Chinese name	Latin name	Family	Scientific name	Plant part	%	Origin (China)
Dihuang	*Radix et Rhizoma Rhei*	Polygonaceae	*Rehmannia glutinosa* (Gaertn.) DC.	Rhizome	37	Si Chuang
Dazao	*Fructus Jujubae*	Rhamnaceae	*Ziziphus jujuba* Mill.	Fruit	14	Hebei
Maidong	*Ophiopogonis Radix*	Liliaceae	*Ophiopogon japonicus* (Thunb.) Ker Gawl.	Root	13	Guang Dong
Zhigancao	*Radix Glycyrrhizae Preparata*	Leguminosae	*Glycyrrhiza glabra* L.	Rhizome	9	Gan Su
Heizhima	*Sesami Semen Nigrum*	Pedaliaceae	*Sesamum indicum* L.	Seed	7	Hu Bei
Guizhi	*Ramulus Cinnamomi*	Lauraceae	*Cinnamomum cassia* (L.) J. Presl	Branch	6	Guang Dong
Shengjiang	*Rhizoma Zingiberis Recens*	Zingiberaceae	*Zingiber officinale* Roscoe	Rhizome	6	Hebei
Ejiao	*Asini Corii Colla*	Equidae	*Equus asinus* L.	Hide gelatin	4	Shan Dong
Hongshen	*Radix Ginseng Rubra*	Araliaceae	*Panax ginseng* CA Mey.	Rhizome	4	Ji Lin

## Data Availability

The data used to support the findings of this study are available from the corresponding author upon request.

## Abbreviations

CK: Creatine kinase
CVD: Cardiovascular disease
DHE: Dihydroethidium
ECG: Electrocardiogram
H&E: Hematoxylin and eosin
IC_50_: Concentration required to inhibit transport by 50%
I_Ca-L_: L-type calcium current
IHD: Ischemic heart disease
IL-6: Interleukin-6
LTCC: L-type Ca^2+^ channel
ISO: Isoproterenol
LDH: Lactate dehydrogenase
MDA: Malondialdehyde
MI: Myocardial ischemia
NCX: Na^+^/Ca^2+^ exchanger
ROS: Reactive oxygen species
RyR: Ryanodine receptor
SOD: Superoxide dismutase
TNF-*α*: Tumor necrosis factor-*α*
TCM: Traditional Chinese medicine
TEM: Transmission electron microscope
YXDJ: YangXinDingJi capsule.
